# Books and Authors

**Published:** 1906-08

**Authors:** 


### BOOKS AND AUTHORS.

**The Influence of the Menstrual Function on Certain Diseases of the Skin.** By **L. Duncan Bulkley,** A.M., M.D., Physician to the New York Skin and Cancer Hospital, Consulting Physician to the New York Hospital, Consulting Dermatologist to the Randall's Island Hospitals, to the Manhattan Eye and Ear Hospital, and to the Hospital for Ruptured and Crippled, etc. Demi-octavo, pp. 108. New York (1123 Broadway) and London: Rebman Company. (Price: $1.00).

The author of this volume is known the world over as one of the leading dermatologists of America and as one who has the faculty of imparting his knowledge of the subject, by tongue or pen, with great perspicuity. He is the author of many monographs as well as treatises relating to diseases of the skin. That phase of the topic dealt with in this monograph is not only novel but most interesting to the gynecologist as well as general practitioner. Bulkley has grouped here a mass of facts that bear on the menstrual cycle which cannot fail to attract attention and must lead to further observations concerning the relationship between menstruation and skin diseases.

The following synopsis by chapters will give the reader a good idea of the way the author handles the subject. In chapter I are found general considerations, and also menstruation as only the culmination of "cyclic changes" in the system, is presented. Then follow changes in the urea, temperature and weight of body, thyroid gland, blood, nervous system, and the like.

In chapter II, clinical records are offered under the following headings: personal instances of the influences of menstruation in certain diseases of the skin; influence of menstruation on acne, eczema, herpes, pemphigus,dermatitis herpetiformis, lichen, urticaria, edema and cutaneous nodes, erythema, erysipelas, ecchymosis and purpura, "bloody sweat," pruritus, psoriasis, dermatitis seborrheica, hyperidrosis, bromidrosis, chloasma, melanoderma, furunculosis, abscess, syphilis, lupus, epithelioma, scleroderma, morphea, hypertrichosis, and the like.

In chapter III, an analysis of facts and theories is recorded. The most plausible theories as to influence, for example, as:

I. Cylical changes in the general system. 2. Auto-intoxication of genital origin. 3. Nervous reflex irritation from congested genital apparatus.

Application of theories to various results in the skin.

Finally in chapter IV, treatment is presented. A bibliography is then given and the volume closes with an excellent index.

---

**On the Nature, Causes, Variety and Treatment of Bodily Deformities.** By the late **E. J. Chance,** F.R.C.S., Eng. Edited by **John Poland,** F.R.C.S., Eng. A series of lectures. Second edition. In two volumes—Vol. I. Duodecimo. pp. 314. London: Smith, Elder & Co., 15 Waterloo Pl., S. W. 1905. (Price, 6s net. $1.50).

Mr. Chance was one of the foremost orthopedic surgeons of his time, and was a pioneer as a specialist in that branch of surgical science, in which he did much original work. The lectures which form the basis of this book were delivered over fifty years ago; the book itself was published first over forty years ago, while the distinguished author has been dead more than ten years. To be a good orthopedic surgeon a man must be a mechanic, a pediatrist, a general surgeon, and, likewise, possess inventive genius. Mr. Chance was all of these and more; he was not only devoted to the science of medicine as a whole, but was broadly trained as a man.

No one can read these lectures without absorbing knowledge. They betray anatomical knowledge, surgical skill, and artistic accomplishment. They deal with the subject differently from the way adopted by most authors, and many of the illustrations are drawn by the author's own hand. The editor, too, has performed a difficult task most admirably, in fashioning the work to meet present conditions.

---

**A Laboratory Manual of Physiological Chemistry.** By **Elbert W. Rockwood,** M.D., Ph.D., Professor of Chemistry and Toxicology and head of the department of Chemistry in the University of Iowa. Second edition. revised and enlarged. With one colored plate and three plates of microscopic preparations. Large 12mo, 229 pages. Philadelphia: F. A. Davis Company. (Price: $1.00 net).

The progress made in the department of biology dealt with under the name of physologic chemistry has been very considerable during the last seven years, or since the first edition of this manual was sent out. A new edition, therefore, became imperative in order to present the subject in its best light and in proper form. The same general plan has been adhered to as in the original, the changes being principally such as further experience has suggested. It is essentially a book for the student to use during his laboratory studies, but is equally useful to the general practitioner who is wont to study his cases in the light of clinical laboratory investigation. To all such we most heartily commend it.

---

**Transactions of the American Surgical Association for the Year 1905.** Volume XXIII. Edited by **Richard H. Harte.** M.D., recorder of the Association. Printed by the Association. William J. Dornan, Philadelphia, 1905.

This volume contains the record of the work of the association at its San Francisco meeting held July 5-7, 1906, under the presidency of Dr. George Ben Johnston, of Richmond. The subject of the presidential address is "John Peter Mettaner," and is a biographic sketch of this distinguished and somewhat eccentric physician, who practised medicine and surgery in Prince Edward County for nearly three-quarters of the nineteenth century. The story is told in Dr. Johnston's peculiarly graceful fashion, and furnishes charming entertainment for a leisure hour.

Many papers of surgical interest and importance go to make up a valuable book, only two of which, for lack of time and space, may we mention. Dr. George Ryerson Fowler presented the history of a case of suture of the spinal cord for gunshot injury causing complete severance of the cord. Twenty-six months after the operation the patient was living, but voluntary motion in the affected area was practically lost. Dr. Rudolph Matas gave his further experiences in the radical operation for the cure of aneurysm, by his own method of intrasaccular suture. A group of 27 cases was reported from all operators, without fatal results.

